# Group-based activities with on-site childcare and online support improve glucose tolerance in women within 5 years of gestational diabetes pregnancy

**DOI:** 10.1186/1475-2840-13-104

**Published:** 2014-06-30

**Authors:** Anne-Sophie Brazeau, Aaron Leong, Sara J Meltzer, Rani Cruz, Deborah DaCosta, Mary Hendrickson-Nelson, Lawrence Joseph, Kaberi Dasgupta

**Affiliations:** 1 Division of Clinical Epidemiology, Department of Medicine, McGill University Health Centre, 687 Pine Avenue West, H3A 1A1, Montreal, Quebec, Canada

**Keywords:** Gestational diabetes, Type 2 diabetes, Prevention, Insulin resistance, Diet, Exercise, Childcare, Spouse participation

## Abstract

**Background:**

Women with gestational diabetes history are at increased risk for type 2 diabetes. They face specific challenges for behavioural changes, including childcare responsibilities. The aim of this study is to test a tailored type 2 diabetes prevention intervention in women within 5 years of a pregnancy with gestational diabetes, in terms of effects on weight and cardiometabolic risk factors.

**Methods:**

The 13-week intervention, designed based on focus group discussions, included four group sessions, two with spousal participation and all with on-site childcare. Web/telephone-based support was provided between sessions. We computed mean percentage change from baseline (95% confidence intervals, CI) for anthropometric measures, glucose tolerance (75 g Oral glucose tolerance test), insulin resistance/sensitivity, blood pressure, physical activity, dietary intake, and other cardiometabolic risk factors.

**Results:**

Among the 36 enrolled, 27 completed final evaluations. Most attended ≥ 3 sessions (74%), used on-site childcare (88%), and logged onto the website (85%). Steps/day (733 steps, 95% CI 85, 1391) and fruit/vegetable intake (1.5 servings/day, 95% CI 0.3, 2.8) increased. Proportions decreased for convenience meal consumption (−30%, 95% CI −50, −9) and eating out (−22%, 95% CI −44, −0) ≥ 3 times/month. Body mass index and body composition were unchanged. Fasting (−4.9%, 95% CI −9.5, −0.3) and 2-hour postchallenge (−8.0%, 95% CI −15.6, −0.5) glucose declined. Insulin sensitivity increased (ISI _0,120_ 23.7%, 95% CI 9.1, 38.4; Matsuda index 37.5%, 95% CI 3.5, 72.4). Insulin resistance (HOMA-IR −9.4%, 95% CI −18.6, −0.1) and systolic blood pressure (−3.3%, 95% CI −5.8, −0.8) decreased.

**Conclusions:**

A tailored group intervention appears to lead to improvements in health behaviours and cardiometabolic risk factors despite unchanged body mass index and body composition. This approach merits further study.

**Clinical trial registration:**

ClinicalTrials.gov (NCT01814995).

## Introduction

Women with a gestational diabetes (GDM) history have a greater than seven-fold risk increase for type 2 diabetes [1,2] and increased cardiovascular disease risk [3,4], compared to women without glucose elevations in pregnancy. The American Diabetes Prevention Program trial (DPP) [5] demonstrated that in prediabetes, an individualised eating and physical activity behaviour change program led to a greater than 50% reduction in type 2 diabetes incidence. Further, impact was specifically confirmed in women within an average of 12 years of first GDM pregnancy. However, given that the period of highest diabetes incidence is within 5 years of a GDM pregnancy [6], the DPP intervention was arguably “late.” Engaging women earlier could not only prevent more cases of type 2 diabetes but also reduce the risk of recurrent GDM [7]. Notably, subsequent pregnancies without GDM are an indicator of decreased risk of type 2 diabetes development later in life [8].

Unfortunately, trials within 5 years of a GDM pregnancy have met with limited success. Interventions have mainly been at the individual level, including dietary and physical activity counselling and support. These have been delivered through face-to-face consultations with additional phone/text/email support [9-12] or without face-to-face contact, relying exclusively on phone calls and written material [13] or web-based programs [14]. A single study examined a group-based approach consisting of weekly physical activity sessions [15]. In all cases, impact on weight was limited [10-14]. When assessed, no improvements in fasting [10,12,14,15] or 2-hour post challenge glucose [12,14,15] have been observed. Further, participation appears to be challenging [15,16], largely because of work and family-related responsibilities. There is a need for the development and testing of alternate strategies, as well as assessment of impact on a greater variety of cardiometabolic risk factors, to comprehensively capture possible benefits.

As previously reported, we conducted focus group discussions with women within 5 years of a GDM pregnancy, seeking their input on the design of a realistic intervention strategy. Our analyses signalled a need for group-based face-to-face interactions with peers and professionals [17]. However, to facilitate attendance, on-site childcare was viewed to be important. Phone, web-based, and text support were considered adjunctive. Spousal involvement was deemed critical to allow health behavioural change at home. We designed an intervention that incorporated these elements. We assessed its impact through the MoMM pilot study, evaluating not only changes in body weight but in insulin resistance, blood pressure, lipid profile, eating behaviours and daily step counts, as reported herein.

### Subjects and methods

MoMM was a single-arm pilot interventional study (ClinicalTrials.gov: NCT01814995) examining pre- to post intervention changes. This design permits each woman to act as her own control [18]. The protocol was approved by McGill University’s Faculty of Medicine Institutional Review Board and all participating institutions (McGill University Health Centre, Sir Mortimer General Jewish General Hospital, and Concordia University). Participants provided written informed consent. The acronym MoMM originally stood for *Combining* MO*tivational Support,* M*eal Preparation Training, and a Tapering Course of* M*eal Replacements To Achieve Vascular Risk Reduction in Women with a Gestational Diabetes History*; however, focus group discussion indicated that women preferred not to use meal replacements. The title was therefore changed to M*indful m*O*vement,* M*indful eating,* Mi*ndful living*.

### Eligibility criteria and recruitment

Inclusion criteria were GDM pregnancy within the prior 5 years and BMI ≥ 24 kg/m^2^. Exclusion criteria were other forms of diabetes, current use of antihyperglycemic medication, pregnancy or attempting to become pregnant, current smoking, or co-morbid conditions or medications that could impact weight. Recruitment occurred through the GDM clinics of participating institutions. Strategies included invitation letters from the treating physicians with follow-up telephone calls by study personnel (Additional file 1: Figure S1).

### Intervention

There were four monthly group-based sessions, each 4 hours in duration and held on a weekend day morning at Concordia University’s PERFORM centre (http://performcentre.concordia.ca/en/), a research and teaching facility equipped with a kitchen (four work stations) and an exercise area. Partners were invited to sessions 2 and 4. Experienced childcare providers from a nearby daycare were hired to supervise the children on-site during sessions.

Sessions began with a one-hour period with an exercise physiologist who discussed the importance of physical activity and strategies for its integration into the daily routine. Participants were provided with pedometers and resistance exercise bands. At the first session, they did a practice walk with the pedometer and were instructed in its use. They were encouraged to monitor their daily steps, aiming to progressively achieve/surpass 10,000 steps/day [19]. They practiced floor exercises, with and without the resistance bands, under supervision.

Over the following three hours, a registered dietitian discussed eating behaviour and nutrition and supervised participants in the preparation of a balanced meal. They were encouraged to eat at regular intervals to avoid strong ‘hunger’ sensations that could provoke over-eating; to plan intake; to have healthy snacks visible at home and work (e.g., eat a fruit first); to take the time to savour meals and snacks with minimal distractions to help recognition of satiety signals; and to try to eat as a family. There was an emphasis on appropriate food group proportions demonstrated using the Canadian Diabetes Association's Balanced Plate Method (e.g., ½ plate vegetables, ¼ plate cereals and grains, ¼ plate protein) and the Handy Portion Guide [20]. This was operationalized when participants and their families served themselves the meal, under the dietitian’s supervision. On a separate occasion, participants had an opportunity to meet with a nutrition student for a grocery store tour, to discuss produce selection.

The study-specific website included information on GDM and type 2 diabetes, recipe ideas, stress management tips, and links to tools for the tracking of dietary intake (http://www.eaTracker.ca) and physical activity (http://www.stepscount.com). A discussion forum was available to address barriers to achieving healthier choices. Participants were also contacted individually by telephone two to three times per month by study personnel, to assess progress and to respond to any questions.

### Outcomes

Demographic information, family history, and past medical history were queried at baseline. All other assessments were conducted both at baseline and post intervention at the McGill Nutrition and Performance Laboratory, following an overnight fast.

#### Anthropometric measures and body composition

Weight to the nearest 0.1 kilogram (Digital Physician Scales, model 140-10-6 by Rice Lake Weighing Systems, light clothing, shoes removed) and height to the nearest 0.1 centimeter (Stadiometer PE-WM-60-76-BRG2, Perspective Enterprises) were assessed and BMI calculated. Waist circumference was measured midway between the iliac crest and the lower rib margin. Dual-Energy X-ray absorptiometry (DXA; GE Lunar Prodigy Advance) was performed to estimate the percentage of total body fat.

#### Oral glucose tolerance test and insulin resistance

Plasma glucose (glucose oxidase method) and insulin (ELISA method) were measured on blood samples drawn in the fasting state and at 60 and 120 minutes following ingestion of a 75 g glucose solution. The Homeostatic Model Assessment Insulin resistance (HOMA-IR) [21], Matsuda index [22,23], and insulin sensitivity index (ISI _0, 120_) [24] were computed. *Other serum markers.* Lipid parameters (Total-cholesterol, HDL, Triglycerides) were measured on fasting blood samples (Piccolo xpress technic) and LDL was calculated. Adiponectin and leptin concentrations were assayed (Human Total Adiponectin/Acrp30 Quantikine ELISA Kit, cat. DRP300, R&D systems; Human Leptin Quantikine ELISA Kit, cat. DLP00, R&D systems). *Blood pressure*. Blood pressure was assessed in a seated position with the arm supported following a 5-minute rest period. Six measurements at 1-minute intervals were recorded (Mindray Accutor –V Vital Signs Monitor). The last 5 systolic (SBP) and diastolic blood pressure (DBP) measurements were separately averaged.

#### Dietary intake and eating behaviour

A registered dietitian conducted 24-hour dietary recalls to determine number of daily servings by food group (Canada Food Guide) [25]. Participants were asked about the frequency of eating pre-prepared convenience foods or restaurant meals [26]. Perceived ability to cook from basic ingredients was assessed (7-point scale) [27]. Other self-administered questionnaires included the Weight Efficacy Lifestyle (WEL) [28] which assesses eating-related self control (scores 1–9) and the Mindful Eating Questionnaire (MEQ) [29] (scores 1 to 4). The Weight Stages of Change-Short Form was also administered [30].

#### Physical activity behaviour

Daily step counts were measured using Yamax SW-200 (Yamasa Tokei Keiki Co., Ltd, Tokyo, Japan) pedometers over 7 days [31]. Participants also wore a multi-axial accelerometer (ActiGraph GT3X, Actigraph LLC., Pensacola, FL). To be included in the analysis, participants had to have worn the accelerometer for at least 10 hours per day for a minimum of 3 days. *Other.* Participants completed the Hospital Anxiety and Depression Scale (HADS, 14 items) [32]. At the time of the final evaluation, participants completed a questionnaire querying utility of the sessions, with specific questions about the cooking and physical activity components, the importance of on-site childcare, reasons for partner’s attendance/non-attendance, utility of the eaTracker tool, pedometer, pedometer step count tracker tool, and MoMM website, as well as suggestions about ways to improve the website.

### Statistical analysis

Descriptive statistics are presented as means and standard deviations (SD), median and interquartile range (IQR), or proportions, as appropriate. Mean changes and 95% confidence intervals were calculated in unit and percentage change from baseline for clinical and behavioural parameters.

## Results

Thirty-six women were recruited. Among these, 27 (75.0%) completed baseline and final assessments. Among the 9 women that withdrew, 5 did not attend any group-sessions (new pregnancy, 2; divorce or death in family, 2; unspecified, 1) and 4 attended at least one (time pressure, 2; feeling depressed, 1; divorce, 1). Those who completed both assessments were, on average, approaching their middle years (mean age 40 years, SD 5) and educated beyond high school (88.9%). More than half (63.0%) were Europid, most lived with their partner (92.6%), had an average of two children, and worked outside the home (74.1%). Questionnaire data indicated that 5 (18.5%) had elevated anxiety levels and one had depressed mood (3.7%). Roughly half reported limited social media use (never, 18.5%; monthly, 33.3%). At baseline, most were in an action (44.4%) or maintenance (18.5%) stage for weight change.

Four women (14.8%) were included in the study although their BMIs were < 24 kg/m^2^, (i.e., did not meet eligibility criteria). We opted to include them because of recruitment challenges. Three (11.1%) more had a BMI between 24 and 25 kg/m^2^, 10 (37.0%) were overweight (25 ≤ BMI < 30 kg/m^2^) and 10 (37.0%) were obese (BMI ≥ 30 kg/m^2^). Eleven (37.0%) met criteria for dysglycemia (3 impaired fasting glucose alone, 1 impaired glucose tolerance alone, 6 impaired fasting glucose and impaired glucose tolerance, 1 type 2 diabetes). We opted to retain the individual with type 2 diabetes but performed a sensitivity analysis excluding this individual.

Roughly one third (33.3%, Table 1) who completed baseline and final assessments attended all in-person sessions. Approximately half (48.1%) missed a single session. Most used the free on-site childcare (88.9%) at all sessions that they attended. Among the 25 (92.6%) who lived with a partner, 10 (40.0%) had a partner who attended at least one session. Almost all participants (85.2%) visited the MoMM website. Half (51.9%) used the on-line step count-tracking tool and logged onto the eaTracker tool (59.3%). Participants emailed the staff-contact a median of 5.5 times (IQR 5.5-15.5) over the course of the study.

**Table 1:** Participation during the 13-week intervention

**Intervention components**	
*Sessions attendance*	
Four sessions; n (%)	9 (33.3%)
Three sessions; n (%)	11 (40.7%)
Two sessions; n (%)*	6 (22.2%)
*Child-care use*	
Ever use; n (%)	24 (88.9%)
Sessions used among those with ever use (% of attended sessions)	100%
*Partners' attendance*^†^	
One session; n (%)	7 (28.0%)
Two sessions; n (%)	3 (12.0%)
*Grocery store visit*	
Signed up to participate; n (%)	7 (25.9%)
Participated; n (%)	2 (7.4%)
*Web-use*	
Visited website; n (%)	23 (85.2%)
Visits to website, median (IQR)	21 (8.4-44.5)
Use of step count log; n (%)	14 (51.9%)
Use of eaTracker; n (%)	16 (59.3%)
Visited web-forum; n (%)	13 (48.1%)
Posted comments on web-forum; n (%)	2 (7.4%)
*Contact by Participants with MoMM coordinator*	
Contacted by email; n (%)	25 (92.6%)
Emails sent by participants; median (IQR)	5.5 (5.5-15.5)
Contacted by text messages; n (%)	4 (3.7%)

*Two women were recruited after the first group session; they participated in two out of three sessions.

^†^Two women were single parents; Maximum of partners that could attend = 25.

### Dietary intake, eating behaviours, and physical activity

**Figure 1:**
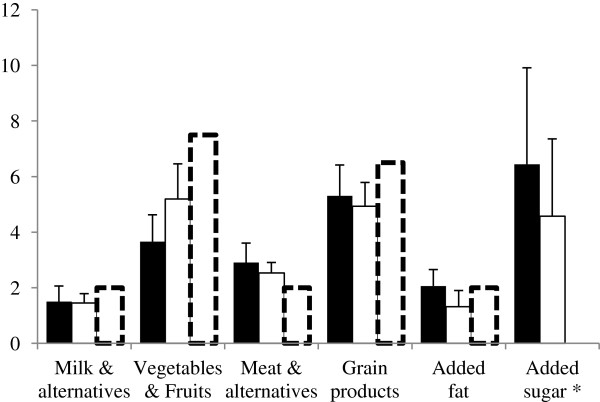
**Daily servings compared to the Canada’s Food Guide.** Data are means and 95% Confidence intervals. Black bars = baseline values; White bars = final values; Hatched white bars = Canada Food Guide recommendation. * A portion of added sugar = 5 g of carbohydrate.

Most (n = 19; 73%) perceived themselves to be at least a competent cook at baseline. This increased to 77.8% (n = 21) post intervention. Fruit and vegetable consumption increased from 3.7 (SD 2.7) servings/day at baseline to 5.2 (SD 3.4) at the final evaluation (mean increase: 1.5 servings/day, 95% CI 0.3, 2.8) (Figure 1). The proportion of participants consuming pre-prepared convenience meals more than three times per month declined from 48% at baseline to 19% post intervention (−30%, 95% CI −50, −9). The proportion reporting eating out more than three times per month decreased from 50% at baseline to 30% post intervention (−22%, 95% CI −44, 0). There were no important changes in the WEL or MEQ scores. There were no important changes in accelerometer-based measures of physical activity, but only 20 participants wore their accelerometer the required ≥ 10 hours per day for at least during 3 days. In contrast, pedometer data indicated that by the end of the program, women were completing an additional 733 steps/day (95% CI 85, 1391) reaching a mean of 7,762 (SD 2368). This change corresponds to an 18.8% improvement (95% CI 6.6, 20.9).

### Weight and body composition

**Figure 2:**
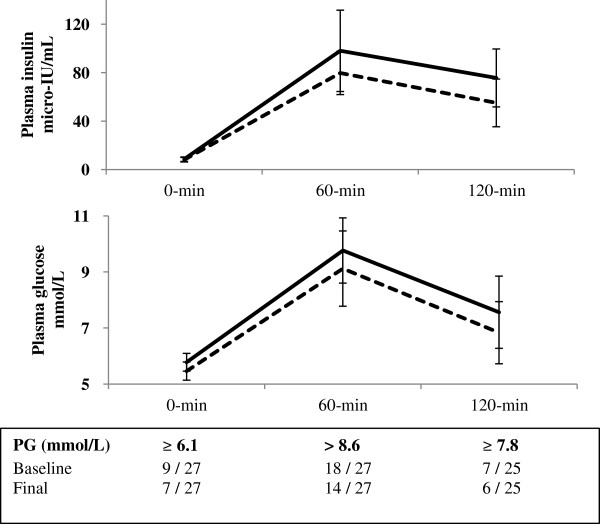
**Plasma glucose and plasma insulin during the oral glucose tolerance tests.** Black line = baseline; Hatched line = final. PG = Plasma glucose. Data are means and 95% confidence intervals.

The mean weight loss (Table 2) was −0.2 kg of total body weight (95% CI −1.0, 0.5) and −0.1 kg of total fat mass (95% CI −0.8, 0.5). *Glucose tolerance*. There were mean reductions in FPG (−0.3 mmol/L, 95% CI −0.6, −0.0) and in 2hPG (mean −0.7 mmol/L, 95% CI −1.4, −0.1) values (Table 2; Figure 2). The participant with a new diagnosis of type 2 diabetes had reductions in both FPG, from 8.5 to 5.2 mmol/L, and in 2hPG, from 16.5 to 11.2 mmol/L, without institution of any antihyperglycemic medication. *Measures of insulin resistance and sensitivity*. Insulin resistance, as captured by HOMA-IR, decreased by a mean of −0.2 (95% CI −0.5, 0.0), nearly a 10% reduction (−9.4%, 95% CI −18.6, −0.1). Correspondingly, there were increases in both measures of insulin sensitivity. The Matsuda index increased by a mean of 1.5 (95% CI −0.3, 3.3), a 37.9% increase (95% CI 3.5, 72.4). The ISI_0,120_ increased by a mean of 13.1 mg × L^2^/mmol × μU × min (95% CI 5.0, 21.2), a 23.7% increase (95% CI 9.1, 38.4). Women in all BMI categories demonstrated a similar trend for glucose tolerance change (Additional file 1: Table S1). In a sensitivity analysis excluding the individual with type 2 diabetes retained, the point estimates were unchanged but the confidence intervals slightly widened (e.g. 2hPG went from −8.0% [95% CI −15.6, −0.5] to −7.6% [95% CI −14.6, 0.5] and ISI_0,120_ from 23.7% [95% CI 9.1, 38.4] to 22.4% [95% CI 7.4, 37.4)]. *Blood pressure*. There were reductions in both SBP (−4.2 mmHg, 95% CI −7.4, −1.0) and in DBP (−3.1 mmHg, 95% CI −5.3, −0.9). *Lipid profile*. Triglycerides were also lower post intervention (−0.4 mmol/L, 95% CI −0.4, −0.1), but other lipid parameters (HDL, LDL and Total-Cholesterol) remained unchanged. *Adiponectin and leptin*. Changes for adiponectin and leptin values were inconclusive with large confidence intervals.

**Table 2:** Changes in cardiometabolic measures

	**Baseline all (n = 36), mean (SD)**	**Baseline completed (n = 27), mean (SD)**	**Final completed (n = 27), mean (SD)**	**Change from baseline, mean [95% CI]**
Weight, kg	77.1 (19.0)	76.4 (17.1)	76.2 (17.2)	−0.3% [−1.3, 0.7]
Body mass index, kg/m^2^	29.3 (7.0)	29.1 (6.7)	29.0 (6.8)	−0.3% [−1.3, 0.7]
Waist circumference, cm	92.7 (14.6)	91.8 (13.7)	90.8 (13.6)	−0.9% [−2.6, 0.7]
Fat mass, %*	40.7 (7.1)	41.7 (6.7)	41.5 (7.0)	−0.5% [−2.0, 1.1]
Systolic blood pressure, mm Hg	119.6 (11.2)	120.0 (11.2)	115.8 (11.4)	−3.3% [−5.8, −0.8]
Diastolic blood pressure, mm Hg	70.4 (6.7)	71.3 (6.9)	68.2 (8.4)	−4.3% [−7.3, −1.3]
Fasting plasma glucose, mmol/L	5.7 (0.8)	5.8 (0.8)	5.5 (0.9)	−4.9% [−9.5, −0.3]
1-h plasma glucose, mmol/L	9.5 (2.9)	9.8 (3.1)	9.1 (3.6)	−5.0% [−19.1, 9.1]
2-h plasma glucose, mmol/L^†^	7.2 (2.9)	7.6 (3.3)	6.8 (2.8)	−8.0% [−15.6, −0.5]
Fasting insulin, μU/mL	7.9 (4.5)	8.5 (4.9)	8.1 (5.0)	−5.3% [−13.3, 2.7]
1-h insulin, μU/mL*	88.7 (80.0)	101.7 (88.8)	82.5 (46.3)	−2.2% [−24.9, 20.6]
2-h insulin, μU/mL^†^	63.5 (65.0)	75.7 (72.7)	55.0 (50.2)	−15.9% [−35.6, 3.8]
ISI _0,120_, mg × L^2^/mmol × μU × min^†^	56.6 (26.3)	59.3 (31.9)	72.3 (41.4)	23.7% [9.1, 38.4]
Matsuda Index^†^	5.6 (3.9)	5.0 (4.0)	6.5 (6.4)	37.9% [3.5, 72.4]
HOMA-IR	2.0 (1.3)	2.2 (1.4)	2.0 (1.3)	−9.4% [−18.6, −0.1]
Total Cholesterol, mmol/L	5.0 (0.7)	4.9 (0.6)	4.8 (0.7)	−1.9% [−4.9, 1.1]
HDL-cholesterol, mmol/L	1.3 (0.3)	1.3 (0.3)	1.3 (0.4)	0.0% [−4.0, 4.0]
LDL-cholesterol, mmol/L	3.0 (0.6)	2.9 (0.6)	2.9 (0.5)	2.1% [−2.4, 6.7]
Triglycerides, mmol/L	1.4 (0.8)	1.5 (0.9)	1.30 (0.7)	−9.7% [−20.2, 0.9]
Adiponectin, μg/mL	6.8 (4.4)	6.6 (4.0)	6.7 (4.0)	2.2% [−3.7, 8.1]
Leptin, ng/mL	25.2 (16.7)	23.6 (13.8)	26.2 (17.5)	11.4% [−1.8, 24.6]

*Data available for 26 who completed baseline and final assessments.

^†^Data available for 25 who completed baseline and final assessments.

### Participants’ impressions of the program

Twenty-three participants completed the post intervention questionnaire related to impressions/opinions related to the intervention strategy. There was strong indication that participants perceived sessions as useful in promoting eating behaviour change (92%). The incorporation of ‘hands-on’ cooking was deemed important (88%) and indication that the physical activity education sessions helped increase activity levels (92%). A large proportion indicated that the pedometer was a useful tool (92%). Many noted that the availability of on-site childcare facilitated session attendance (79%). In terms of partner attendance, 9% did not have a partner and 70% indicated that their partner could not attend (35%- no time; 35%- did not want to attend).

In terms of on-line tools, over half (56%) indicated that the MoMM website was useful to them, 44% reported the on-line step count log to be helpful, and 40% appreciated the eaTracker tool. Conversely, an important proportion reported never logging on to these sites/tools (32% for MoMM website, 40% for on-line step count log, 36% for eaTracker). Some individuals did log on but did not perceive the tools to be useful (12% for MoMM website, 16% for on-line step count log, 24% for eaTracker); these individuals either experienced frustrations/challenges with the sites (amount of information, errors, juggling passwords- four responses), preferred to use paper and pencil tracking (three responses), or may have preferred a *Facebook* page (one comment).

## Discussion

Among women within 5 years of a GDM pregnancy who participated in a 13-week tailored intervention (i.e., 4 group sessions, partners invited to two, on-site childcare, meal preparation, pedometers and floor exercises), attendance at sessions was high, the childcare service and website were used, and there was some spousal participation. Self-reported eating control was not enhanced and there were no changes in BMI or body composition. However, there were important increases in fruit and vegetable intake and consumption of both convenience meals and eating out declined. Step counts also increased. There were reductions in both fasting and 2-hour postchallenge glucose and improvements in all measures of insulin resistance and sensitivity. There was also lowering of both systolic and diastolic blood pressure and triglycerides. On balance, the tailored intervention demonstrated both behavioural and biological impact and thus merits further study and development.

Less than a fifth of the participants missed 2 or more of the 4 sessions. On-site childcare may have been a key facilitating factor as evidenced by high use, with almost 90% of the women using it at every session they attended. Further, monthly sessions may have also enhanced attendance, being realistically aligned with time availability. Indeed, in a comprehensive lifestyle change study post pregnancy in a non GDM population, participants were able to attend only 4 of 10 scheduled sessions [16]. Higher attendance had been observed in studies with phone-based or at home sessions, and if sessions are scheduled at the same time of routine medical follow-up [33,34]. For example, among women with GDM who were recruited during pregnancy, 79% completed, during pregnancy, ≥ 2 out of 3 sessions (i.e., 1 in-person and 2 telephone counselling calls) and, in the postpartum period, they participated in an average of 9.4 sessions out of a maximum of 15 (i.e., a maximum of 2 in-persons and a maximum of 13 telephone counselling calls) [9]. We would note, however, that our participants expressed strong endorsement of in-person group sessions and had high levels of attendance.

In the focus group discussions that helped us to design the intervention [17], women expressed a need for spousal involvement. A need for spousal involvement to achieve changes in health behaviour has also emerged in other qualitative studies conducted among women with a GDM history [35,36]. Spousal participation was not a component of previous intervention studies [9-13,15]. The attempt to involve spouses is a novel aspect of our intervention*.* While we did invite spouses to two of the four intervention sessions and there was some participation, a large proportion of spouses did not attend any sessions. Better engagement may further enhance health behaviour change. While time constraints are a real challenge, lack of interest could potentially be addressed with better knowledge about personal and familial diabetes risks related to eating and physical activity habits of the family. Our recent systematic review and meta-analysis [37] estimated that type 2 diabetes in one spouse is associated with a 26% increased risk for type 2 diabetes in the other; further, studies that performed blood tests systematically indicated a doubling of diabetes risk. The concept of shared diabetes risk may potentially be leveraged to increase engagement of spouses in diabetes prevention efforts.

Our post-intervention questionnaire related to impressions/opinions on the intervention strategy suggests a high level of endorsement for in-person, hands-on components, and pedometer use. The availability of on-site childcare was a clear facilitator. While a substantial proportion did use the on-line tools and website, this was much less than in-person session participation. This may not be surprising given that half of participants reported not using web-based media frequently (i.e., less than monthly) at baseline. Web-based tools appear to be underutilised by this group of adults. In a web-based pedometer intervention in women with a GDM history, Kim and colleagues [14] noted that only 3 out of 21 participants used the web forum and questions were only directed to the study staff. This is unfortunate given the low cost and convenience of web and text-based communications. Future studies may need to incorporate strategies to facilitate web-based communication and engagement. For example, study personnel may need to proactively encourage web-based discussion among participants.

The 1.5 servings/day increase in fruit and vegetable intake that we observed likely contributed to the cardiometabolic benefits realized (i.e., glucose, insulin resistance and sensitivity, triglycerides, blood pressure). In a cross-sectional study on 2,115 adults at risk for diabetes, a two-serving increase in daily fruit and vegetable consumption was associated with a 0.08 mmol/L reduction in FPG and 0.2 mmol/L reduction in 2hPG [38]. In a pooled analysis of three prospective cohort studies, fruit consumption was associated with a lower risk of type 2 diabetes (Hazard Ratio 0.98, 95% CI 0.97, 0.99 for three servings/week increment of total whole fruit consumption) [39]. Consistent with the protective effects of fruits and vegetables, diabetes risk calculators, such as FINDRISC, include daily vegetables, fruits and berries consumption as a factor that protects against diabetes development [40]. Moreover, specifically among women, the European Prospective Investigation into Cancer and Nutrition study demonstrated that a 0.5 serving increase in daily intake of vegetables to be associated with 0.20 and 0.09 mm Hg reductions in SBP and DBP, respectively [41]. As reviewed in a meta-analysis of randomised controlled trials, a 10 mm Hg reduction in SBP or 5 mm Hg reduction in DBP leads to a 22% reduction in coronary heart disease events and a 41% reduction in stroke [42]. Our participants achieved mean reductions of more than 4 mm Hg in SBP and 3 mm Hg in DBP, clinically-important changes.

We observed a 733 steps/day increase, greater than that observed in a 12-week web-based pedometer program (mean 543, SD 2074) in women with a GDM history [14]. Among NAVIGATOR trial participants (Nateglinide and Valsartan in Impaired Glucose Tolerance Outcomes Research), both step counts at baseline and mean 6-year increases were associated with reductions in occurrence of cardiovascular events [43]. Our previous studies have demonstrated inverse relationships between blood pressure and step counts, particularly in women [44]. The increase in step counts thus likely also contributed to the cardiometabolic improvements we observed in the present study.

The mean improvements that we observed in FPG and 2hPG are at least as great as those observed in the Finnish Diabetes Prevention trial (−0.3 mmol/L FPG; −0.8 mmol/L 2hPG) [45]. Given that the Finnish Diabetes Prevention Trial, like the DPP, achieved major reductions in diabetes incidence through a lifestyle intervention, the comparability of the improvement in 2-hour post 75 g glucose challenge that we observed appears promising. Further, computations of insulin resistance and sensitivity derived from glucose and insulin measurements consistently demonstrated improvements (HOMA-IR, ISI_0,120_, Matsuda index). Notably, higher insulin sensitivity estimated with the Matsuda index is associated with a decreased incidence of diabetes in high-risk populations [46].

We acknowledge several limitations. First, the primary outcome of our study was a change in weight but no important weight reduction was achieved. More emphasis on energy expenditure reduction may have led to greater weight changes, although adherence may have been challenging. Although our target population was overweight women within 5 years of a GDM pregnancy, some of the women enrolled had a normal BMI. Nonetheless, the BMI range of participants enrolled was more representative of women with GDM in general [47]. Weight reduction observed in overweight/obese women alone was also modest −0.5% (95% CI: −1.7, 0.8), and other changes observed in normal-weight weight women were similar to those observed in the rest of the participants (Additional file 1: Table S1). Second, recruitment was challenging, as anticipated. We endeavoured to recruit women within 5 years of a GDM diagnosis. This is a period of high risk for conversion to type 2 diabetes [6]. While interventions even closer to the time of GDM diagnosis may have had even greater potential impact [6], it would have rendered recruitment more challenging. As reported in our previous focus group study, more than 1,000 invitation letters were sent to women with a prior GDM history. From this pool, we were able to enroll 29 in our focus group study and 28 in the intervention study, with the remainder recruited through telephone contact. The women enrolled may thus not be representative of all women within 5 years of a GDM pregnancy, although they may be representative of those willing to engage in prevention efforts. For capturing dietary intake, the use of a single 24-hour dietary intake recall limits the analysis of macronutrients and micro-nutrients intake (e.g., sodium) that may have impacted cardiometabolic risk factors but does provide an overall view of dietary intake. Accelerometry-based measurements were limited by low wear-time in this cohort. Finally, the lack of a control group is a limitation for this study. A large randomized controlled trial design would be better able to confirm that the cardiometabolic improvements and behavioural changes observed were attributable to the intervention. We plan to conduct such a study.

In summary, the MoMM pilot study indicates that, among women who enroll and participate, a group-based multimodal intervention with childcare support may be effective in lowering diabetes and vascular disease risk in women within 5 years of a GDM pregnancy. Building on such an approach has the potential to reduce diabetes risk and vascular complications- in mothers, fathers, and children.

## Abbreviations

2hPG: 2 hour plasma glucose; DBP: Diastolic blood pressure; DPP: Diabetes prevention program; FPG: Fasting plasma glucose; GDM: Gestational diabetes; HADS: Hospital anxiety and depression scale; IQR: Interquartile range; MEQ: Mindful eating questionnaire; SBP: Systolic blood pressure; SD: Standard deviations; WEL: Weight efficacy lifestyle.

## Competing interests

The authors declare that they have no competing interests.

## Authors’ contributions

ASB, LJ, AL, and KD analyzed data. ASB and KD drafted the manuscript and LJ, AL, DD, SM, MHN, and RC provided important input and revisions. KD, DD, SM, and LJ designed the study. MHN was involved in design of the dietary intervention, AL and RC led recruitment and evaluations and supervised interventions. RC designed the study website in collaboration with KD and the MoMM intervention team, acknowledged above. All authors reviewed and approved the manuscript. The authors have no conflicts of interest. KD had primary responsibility for final content.

## Authors’ information

Anne-Sophie Brazeau and Aaron Leong are Shared 1^st^ author.

## Supplementary Material

Additional file 1: Table S1 Baseline values and percentage of changes from baseline by baseline weight category. **Figure S1.** Recruitment’s flow chart. Click here for file
