# Supplementary material for: Group-based activities with on-site childcare and online support improve glucose tolerance in women within 5 years of gestational diabetes pregnancy
Source: Cardiovasc Diabetol. 2014 Jun 30;13:104. doi: 10.1186/1475-2840-13-104 (PMC4227099; doi:10.1186/1475-2840-13-104)
Supplement: Additional file 1: Table S1 — Baseline values and percentage of changes from baseline by baseline weight category. Figure S1. Recruitment’s flow chart. [file 1475-2840-13-104-S1.pdf]

Supplemental files Table 1 Baseline values and percentage of changes from baseline by baseline weight category

Variables	Baseline BMI < 25 kg/m^2 n=7 mean (SD)	Change from baseline, mean [95% CI]	Baseline 25 ≤ BMI <30 kg/m^2 n=10 mean (SD)	Change from baseline, mean [95% CI]	Baseline BMI ≥30 kg/m^2 n=10 mean (SD)	Change from baseline, mean [95% CI]
Weight, kg	61.5 (7.7)	0.1% [-1.7, 2.0]	70.9 (5.7)	-0.7% [-2.9, 1.5]	92.4 (16.7)	-0.3% [-1.6, 1.2]
Body mass index, kg/m^2	23.1 (1.6)	0.1% [-1.7, 2.0]	26.4 (0.9)	-0.7% [-2.9, 1.5]	36.0 (6.2)	-0.3% [-1.6, 1.2]
Waist circumference, cm	78.2 (8.0)	1.2% [-1.2, 3.6]	90.5 (6.5)	-3.1% [-6.1, -0.0]	102.7 (13.4)	-0.3% [-2.8, 2.2]
Fat mass, %	33.5 (4.7)	2.1% [-5.6, 9.7]	38.0 (5.2)*	-0.9% [-4.2, 2.4]*	47.0 (2.4)	-0.9% [-3.3, 1.6]
Systolic blood pressure, mm Hg	112.0 (10.0)	-5.5% [-9.2, -1.8]	119.7 (7.4)	-1.9% [-6.7, -2.9]	126.0 (12.3)	-3.3% [-7.5, 1.0]
Diastolic blood pressure, mm Hg	68.2 (4.5)	-9.4% [-17.3, -1.5]	71.8 (6.5)	-4.2% [-8.1, -0.4]	72.9 (8.3)	-0.7% [-4.2, 2.8]
Fasting plasma glucose, mmol/L	5.3 (0.6)	-4.3% [-11.5, 3.0]	6.1 (1.0)	-9.6% [-19.8, 0.6]	5.8 (0.6)	-0.6% [-2.1, 3.9]
1-h plasma glucose, mmol/L	6.9 (2.1)	-11.4% [-33.2, 10.3]	10.8 (3.5)	5.1% [-28.2, 38.3]	10.7 (2.0)	-10.5% [-23.3, 2.2]
2-h plasma glucose, mmol/L	5.2 (1.4)	-6.7% [-25.8, 12.3]	9.0 (4.1)	-11.2% [-23.7, 1.3]	7.9 (2.3)	-5.2% [-13.7, 3.3]
Fasting insulin, µU/mL	5.6 (3.3)	-10.2% [-22.4, 2.1]	10.0 (5.5)	-5.3% [-13.3, 2.7]	9.1 (4.8)	-10.4% [-20.7, -0.1]
1-h insulin, µU/mL	70.0 (41.4)	-26.4% [-53.1, 0.3]	121.6 (131.0)	21.3% [-30.7, 73.3]	104.3 (51.7)*	9.4% [-27.6, 8.9]*
2-h insulin, µU/mL	47.2 (49.9)	-21.3% [-71.1, 28.6]	99.6 (103.0)	-10.4% [-42.2, 21.3]	70.6 (26.2)†	-18.1% [-43.5, 7.3]†
$ISI_{0,120}$	74.3 (45.6)	36.9% [-4.2, 77.9]	46.7 (25.1)	24.2% [3.0, 45.3]	61.9 (20.9)†	11.7% [-0.5, 23.9]†
Matsuda Index	8.5 (5.4)	77.4% [-26.6, 181.4]	4.1 (3.0)	26.2% [-18.6, 71.0]	3.2 (1.2)†	18.0% [0.2, 35.9]†
HOMA-IR	1.3 (0.7)	-13.0% [-29.5, 3.6]	2.7 (1.6)	-5.4% [-25.4, 14.6]	2.4 (1.3)	-10.9% [-22.2, 0.4]
Total Cholesterol, mmol/L	4.8 (0.4)	1.7% [-2.8, 6.3]	5.1 (0.8)	-0.1% [-3.7, 3.5]	4.7 (0.6)	-6.2% [-12.0, -0.4]
HDL-cholesterol, mmol/L	1.4 (0.4)	7.5% [0.3, 14.8]	1.2 (0.4)	4.2% [0.9, 7.6]	1.3 (0.2)	-9.4% [-14.6,-4.3]
LDL-cholesterol, mmol/L	2.8 (0.4)	4.2% [-4.8, 13.2]	2.9 (0.7)	4.7% [-3.6, 12.9]	2.8 (0.6)	-1.8% [-8.4, 4.7]
Triglycerides, mmol/L	1.3 (1.1)	-3.2% [-19.8, 13.4]	1.9 (1.0)	-11.0% [-32.9, 10.9]	1.3 (0.6)	-12.8% [-28.3, 2.6]
Adiponectin, µg/mL	10.1 (5.3)	1.4% [-7.2, 9.9]	5.1 (2.3)	5.7% [-3.4, 14.7]	5.5 (2.8)	-0.8% [-12.9, 11.4]
Leptin, ng/mL	13.0 (6.3)	11.4% [-1.8, 24.6]	21.4 (11.7)	14.2% [-16.0, 44.4]	33.3 (13.7)	10.0% [-3.4, 23.3]

*Data missing for 1 participant; †Data missing for 2 participants

Supplemental files Figure 1 Recruitment's flow chart

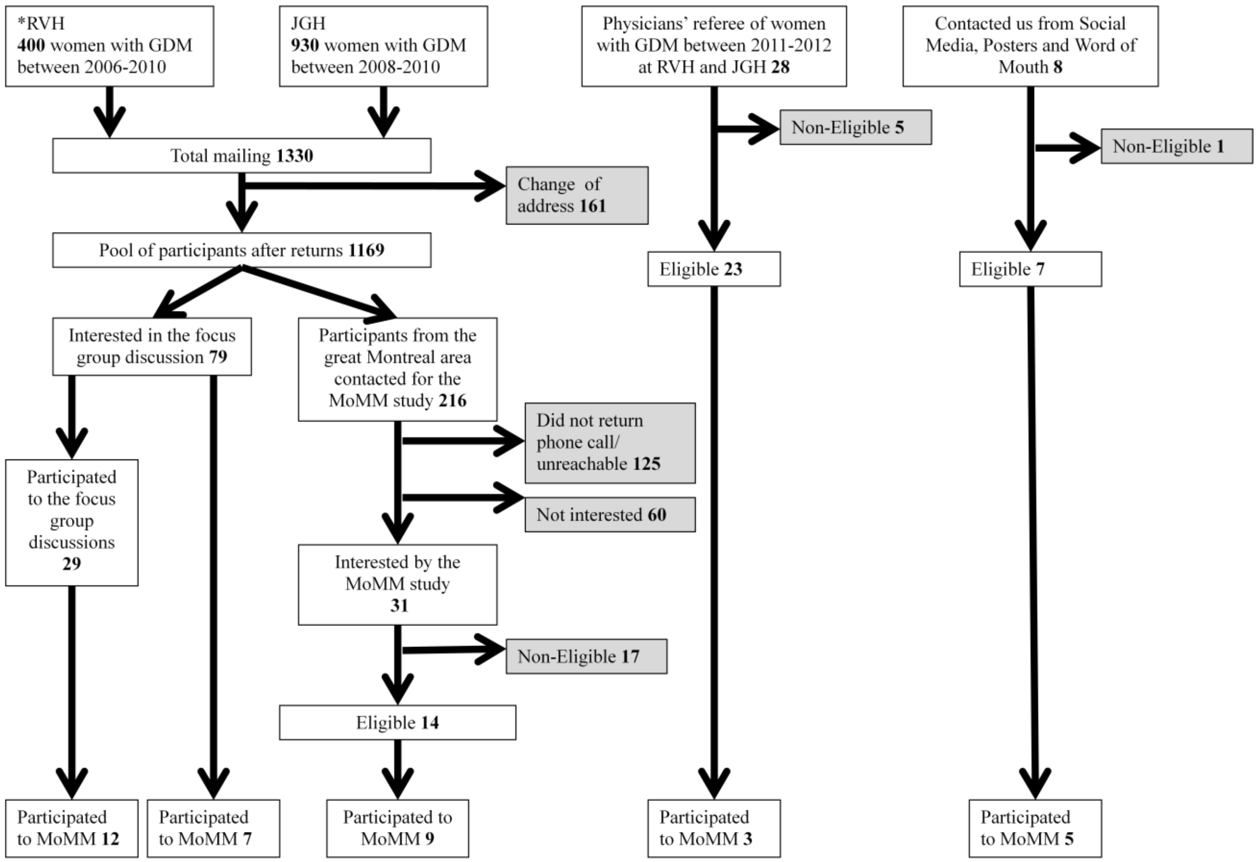

* Only women who presented for post-partum evaluation were included
